# The Effect of Automated Alerts on Provider Ordering Behavior in an Outpatient Setting

**DOI:** 10.1371/journal.pmed.0020255

**Published:** 2005-09-06

**Authors:** Andrew W Steele, Sheri Eisert, Joel Witter, Pat Lyons, Michael A Jones, Patricia Gabow, Eduardo Ortiz

**Affiliations:** **1**Information Services, Denver Health, Denver, Colorado, United States of America,; **2**Health Services Research, Denver Health, Denver, Colorado, United States of America,; **3**General Internal Medicine, Denver Health, Denver, Colorado, United States of America,; **4**Software Division, Siemens Medical Solutions, Malvern, Pennsylvania, United States of America,; **5**Thomson Micromedex, Greenwood Village, Colorado, United States of America,; **6**Medicine, Denver Health, Denver, Colorado, United States of America,; **7**Medicine, Washington D.C. VA Medical Center, Washington, District of Columbia, United States of America; Centers for Disease Control and PreventionUnited States of America

## Abstract

**Background:**

Computerized order entry systems have the potential to prevent medication errors and decrease adverse drug events with the use of clinical-decision support systems presenting alerts to providers. Despite the large volume of medications prescribed in the outpatient setting, few studies have assessed the impact of automated alerts on medication errors related to drug–laboratory interactions in an outpatient primary-care setting.

**Methods and Findings:**

A primary-care clinic in an integrated safety net institution was the setting for the study. In collaboration with commercial information technology vendors, rules were developed to address a set of drug–laboratory interactions. All patients seen in the clinic during the study period were eligible for the intervention. As providers ordered medications on a computer, an alert was displayed if a relevant drug–laboratory interaction existed. Comparisons were made between baseline and postintervention time periods. Provider ordering behavior was monitored focusing on the number of medication orders not completed and the number of rule-associated laboratory test orders initiated after alert display. Adverse drug events were assessed by doing a random sample of chart reviews using the Naranjo scoring scale.

The rule processed 16,291 times during the study period on all possible medication orders: 7,017 during the pre-intervention period and 9,274 during the postintervention period. During the postintervention period, an alert was displayed for 11.8% (1,093 out of 9,274) of the times the rule processed, with 5.6% for only “missing laboratory values,” 6.0% for only “abnormal laboratory values,” and 0.2% for both types of alerts. Focusing on 18 high-volume and high-risk medications revealed a significant increase in the percentage of time the provider stopped the ordering process and did not complete the medication order when an alert for an abnormal rule-associated laboratory result was displayed (5.6% vs. 10.9%, *p* = 0.03, Generalized Estimating Equations test). The provider also increased ordering of the rule-associated laboratory test when an alert was displayed (39% at baseline vs. 51% during post intervention, *p* < 0.001). There was a non-statistically significant difference towards less “definite” or “probable” adverse drug events defined by Naranjo scoring (10.3% at baseline vs. 4.3% during postintervention, *p* = 0.23).

**Conclusion:**

Providers will adhere to alerts and will use this information to improve patient care. Specifically, in response to drug–laboratory interaction alerts, providers will significantly increase the ordering of appropriate laboratory tests. There may be a concomitant change in adverse drug events that would require a larger study to confirm. Implementation of rules technology to prevent medication errors could be an effective tool for reducing medication errors in an outpatient setting.

## Abstract 2

A computerized order entry system that alerted providers to potential problems was shown to be able to influence prescribing practice

## Introduction

Medical error reduction has become a major force for health care in the United States of America since the landmark publication of the 1999 Institute of Medicine report, *To Err Is Human: Building a Safer Health System* and the subsequent report, *Crossing the Quality Chasm: A New Health System for the 21st Century* [1,2]. It is estimated that 700,000 people die or are injured in hospitals each year from adverse drug events (ADEs) [2]. Among the preventable ADEs, more than half were associated with the ordering of the medication [3]. As many as 28% of the ADEs are thought to be associated with medication errors [4]. Computerized Physician Order Entry (CPOE) systems have the capability to address medication errors by utilizing dosing recommendations, reminders for selecting appropriate medications, and clinical checking for drug–allergy, and drug–drug interactions [5–7]. However, despite the potential, less than 10% of hospitals have implemented CPOE [8].

In the outpatient setting, studies have shown that between 18% and 25% of patients may have an ADE [9,10]. Many medications have specific renal, hematologic, and hepatic effects. Monitoring guidelines have been recommended for many of these medications. Health-care providers, however, may find it difficult to acquire and maintain the information to appropriately prescribe these medications. Utilization of a computerized clinical-decision support system linked to patient specific laboratory data within CPOE applications may assist in handling this information overload.

Types of ordering errors include selecting the wrong medication for the patient's illness, choosing an incorrect dose given specific patient characteristics such as age and renal function, and ordering medication when the patient is known to be allergic to the medication. Some errors occur when the provider is not aware of clinical information that is readily available within computer applications [11]. It has been shown that as many as 45% of medication errors may be related to drug–laboratory issues [12]. Further, appropriate monitoring of clinically relevant laboratory values is often inadequate. For example, despite four Federal Drug Administration warnings recommending monthly liver function testing for patients on an oral hypoglycemic, Troglitazone, follow-up studies found less than 5% compliance with these recommendations [13]. Lastly, it is impossible for providers to remember and stay current with all of the new information being provided. For example, the top forty prescribed medications each has on average seven drug–laboratory interactions [14].

This study evaluated the implementation of computerized provider order entry (CPOE) and an integrated computer-based clinical-decision support system (CDSS) in an outpatient clinic at Denver Health as tools for medication error reduction. The study was done in collaboration with Thomson Micromedex and Siemens Medical Solutions. The overarching purpose of the study was to determine the impact of using computerized alerts to improve the prescribing of medications in the outpatient setting. The study focused on drug–laboratory interactions related to medication use that can lead to hyper- and hypokalemia, nephrotoxicity, thrombocytopenia, and hepatic inflammation.

## Methods

### Patients and Setting

This study was conducted at Denver Health outpatient primary-care clinics. Denver Health is the principle safety net institution for Colorado. A single electronic record links primary-care clinics, specialty clinics, and the acute care hospital. Medication and laboratory orders are placed using CPOE. Table 1 provides the demographic characteristics of the patients seen during the study period. There were 19,076 patients seen during this 9-mo time period. Sixty-four percent of the patients were female, 82% were Hispanic, 42% had Medicaid coverage, 41% were uninsured, and 17% had Medicare, private, or other types of insurance.

**Table 1:**
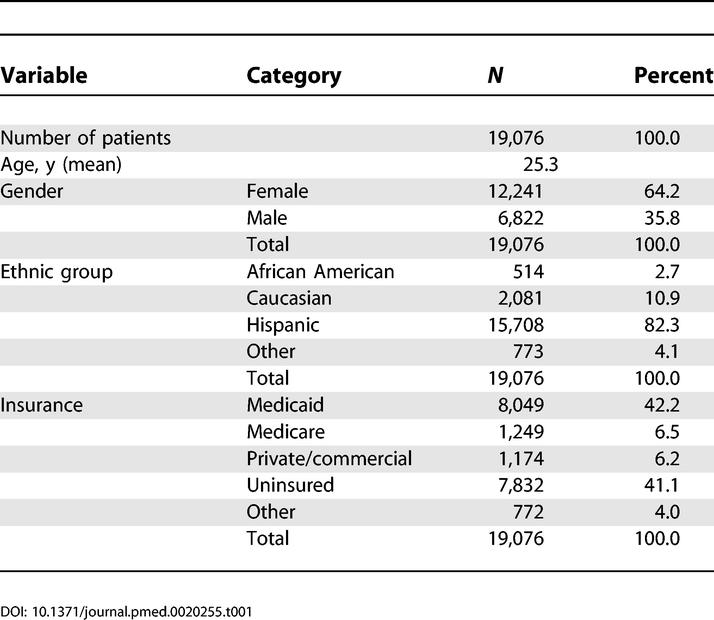
Demographics—All Patients, Westside Clinic (08/01/2002–04/30/2003)

All provider staff were allowed to enter medication orders including physicians, allied health providers (nurse practitioners, physician assistants), and residents. All registered patients were eligible for the intervention.

The Colorado Multiple Institutional Review Board (COMIRB) approved the study. COMIRB is a statewide board established to review biomedical and behavioral research involving humans to ensure appropriate ethical issues are addressed (see Protocol S1).

### Data and Time Frame

Baseline results of rules application were collected from 08/01/2002 to 11/29/2002 (17 wk). The intervention was implemented on 12/01/2002. Postintervention data were collected between 12/01/2002 and 04/30/2003 (21 wk). The timeframe was chosen to provide a sample size of at least 475 which would provide a power (1 − β) of 90% with an α level of 0.05 to detect a difference of 5%, going from 5% to 10% between the order cessation rate comparing preintervention to postintervention phases.

### Interventions

Denver Health has utilized rules technology for over two years. As part of the collaboration with Thomson Micromedex, this study used commercially available rules developed in Arden Syntax language as Medical Logic Modules (MLMs) and modified them to meet the local needs. Although other types of rules were considered, only rules that were commercially available from Thomson Micromedex and applicable to the outpatient setting were used for this study. All rules from Thomson Micromedex were reviewed, and it was determined that the most appropriate available rules to address patient safety in the outpatient setting covered the following five areas: (1) potential drug-induced hypokalemia; (2) potential drug-induced hyperkalemia;(3) potential drug-induced nephrotoxicity; (4) potential drug-induced thrombocytopenia; and (5) potential drug-induced hepatotoxicity.

This study focused mainly on medications that were associated with the above conditions and more commonly used in the outpatient clinic, compared to other medications that were largely used in an inpatient setting. These medications and the associated rules are shown in Table 2.

**Table 2:**
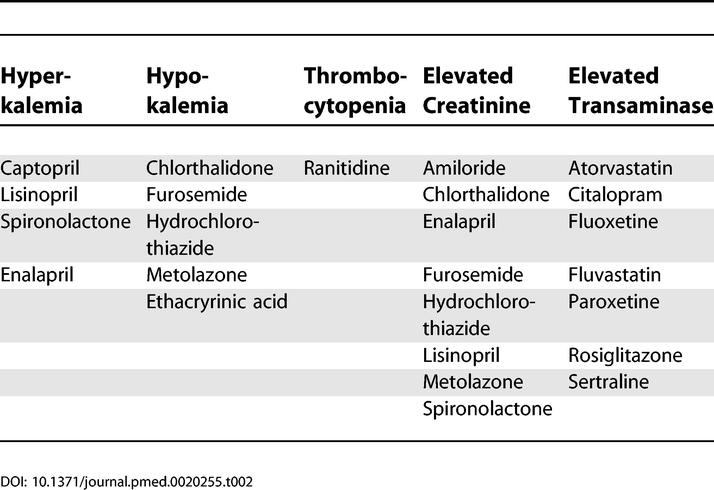
Example of Medications Used in Rules for Associated Lab Abnormalities

For each drug–laboratory interaction, rules were written identifying medications, routes of administration, and abnormal laboratory threshold levels for inclusion in the rule. In addition, a determination was made for each medication as to whether an alert should be provided for an abnormal laboratory value only or either an abnormal laboratory value or a missing laboratory value, or, despite an association with the laboratory abnormality, no alert would be displayed to the provider.

In response to the alerts, providers could decide to keep, revise, or delete the medication order. They could also order any rule-associated laboratory tests. Changes to orders could not be collected given the technological capabilities of the CPOE system, but lack of a medication order after the rule was triggered was evidence that the provider decided to stop the ordering process and not order the medication during that session. Likewise, comparing the rates of ordering of rule-associated laboratory tests before and after the intervention provided a measure of the efficacy of the intervention.

**Figure 1:**
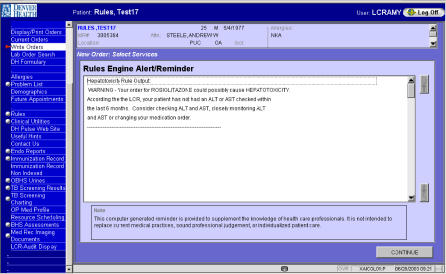
Rules Output Screen for “Recommended Laboratory Value Not Done in Last Six Months”

Thomson Micromedex provided the knowledge content for each of the above rules. One physician (AWS) and a pharmacist (MAJ) utilized a reference THOMSON Micromedex Healthcare Series Integrated Databases and then agreed upon changes to the rules criteria. This was reviewed by a second physician (JW) and some minor changes were subsequently made. The final criteria list was provided to the rules builder staff to incorporate into the rules. The Arden Syntax rules contained within the Medical Logic Modules were integrated into the rules engine application. Due to differences in terminology and unique characteristics of the Denver Health laboratory, order entry, and pharmacy databases, portions of the rules had to be rewritten to conform to these local needs. However, the logic of the rule remained intact. The content and layout of the alert screens were designed by the Information Services staff with input from clinic providers. (Figure 1) The laboratory cutoff values for triggering an alert were the same as the Denver Health abnormal laboratory reference ranges. A timeframe of 6 mo was chosen for using historical laboratory data. Various rules output-message display formats were presented to a group of providers, and consensus was reached on a final display.

During 2002, CPOE was implemented at one of Denver Health's larger outpatient clinics, the Sam Sandos Family Health Clinic. Approximately 120 users were entering over 6,000 orders per week, 40% of which were for medications. As providers selected medications in the ordering process, a rule processed information on the five drug–laboratory interactions listed above. If rule criteria were met for a drug–laboratory interaction, an alert screen was presented to the provider with a message containing patient name, type of rule alert, name of medication that triggered the alert, and a message with laboratory results, if available, and suggestions to consider deleting or changing the medication or to consider ordering a rule-associated laboratory test. Providers did not need to respond to the alert, but needed to select “Continue” to proceed with the ordering session. They were free to make any changes in their ordering process.

No specific provider education was given to the staff concerning the importance and types of drug–laboratory interactions contained within this study.

### Analytical Approach

A nonrandomized pre- and postcomparison of the intervention clinic was accomplished by turning rules on in the background without displaying any message to providers during the preintervention time period. Because the rules were processing in the background, the provider did not receive any alerts recommending changes in their orders. This baseline ordering behavior was then compared to ordering behavior after alerts were presented to the provider.

Medications prescribed and laboratory ordering volume and type were measured at intervention clinics from automated computerized order entry log files. Rules performance was measured by looking at total rules triggered, and further subdividing into rules triggered with no alert provided, rules triggered with alert for missing laboratory tests, and rules triggered with alert for abnormal laboratory values. Provider order behavior was monitored focusing on the number of medication orders not completed after alert display, and the number of rule-associated laboratory test orders initiated after alert display. ADEs were assessed by doing a random sample of chart reviews using the Naranjo scoring scale [15]. The chart audit was limited to a sample of charts for which the most recent rule-associated laboratory value was abnormal within the last 6 mo. The Naranjo criteria has four categories for ADEs; “definite,” “probable,” “possible,” and “doubtful.” This analysis used the combination of “definite” and “probable” as being a potential ADE. Statistical comparisons were made using Fisher's exact test or Generalized Estimating Equations, as appropriate, using a SAS statistical package (SAS Institute, Cary, North Carolina, United States).

## Results

**Figure 2:**
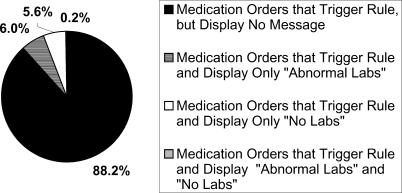
Medication Orders That Triggered Alerts

The rule processed 16,291 times during the study period: 7,017 during the preintervention period and 9,274 during the postintervention period (see Table 3) During the time span of the study there were 54,206 patient visits; medications were ordered on 17,444 (32%) of the visits. The rule processed on 49% of all medication orders. During the postintervention period, an alert was displayed to the care provider for 11.8% (1,093 out of 9,274) of the times the rule processed. Among these alerts, 5.6% were for only “missing laboratory values,” 6.0% were for only “abnormal laboratory values,” and 0.2% were for both types of alerts (see Figure 2). The rule did not have an appreciable negative effect on system performance. On average, the rule delayed processing of the screens in the CPOE application by less than 2 sec. There were no complaints from providers about slow system performance related to the rules processing during this study.

**Table 3:**
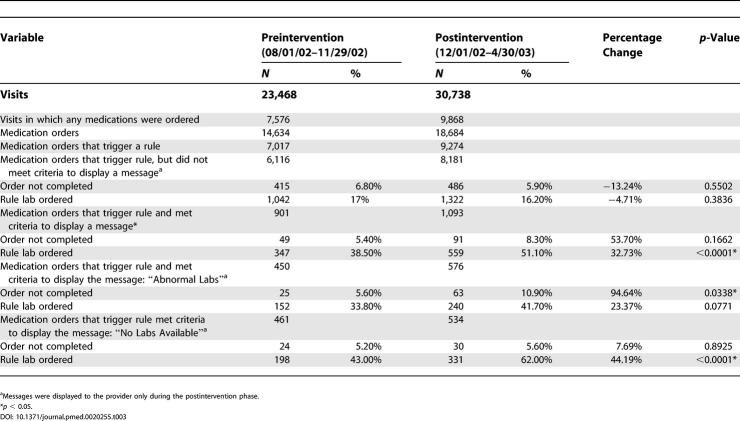
Drug–Laboratory Interaction Rules Performance Summary

Comparing the pre- and postintervention periods for when any type of alert was presented, there was not a statistical difference in the rate of the provider not completing the medication order (5.4% vs. 8.3%, *p* = 0.17). However, when the alert was for an abnormal laboratory value, the percentage of times the medication order was not completed increased from 5.6% at baseline to 10.9% during the intervention (*p* = 0.03).

Comparing the pre- and postintervention periods for medication orders for which no alert was displayed shows no significant change in the percentage of time the provider ordered the rule-associated laboratory test (17.0% during preintervention period vs. 16.2% during the postintervention period, *p* = 0.38). This indicates that there was no trend, in general, to increased laboratory test ordering during the study period. Focusing on medication orders for which an alert was presented shows an increase in the percentage of time the provider ordered the rule-associated laboratory test (38.5% vs. 51.1%, *p* < 0.001). The largest effect was noticed when the alert was triggered for a missing laboratory test: the percentage of times the provider ordered the rule-associated laboratory test increased from 43.0% at baseline to 62.0% (*p* < 0.001).

One investigator (JW) reviewed a random sample of charts. The study focused on medication orders for which an alert was displayed indicating that the patient had a rule-associated abnormal laboratory value. A total of 163 charts were reviewed: 116 from the preintervention period and 47 from the postintervention period (Table 4). Overall, by combining “definite” and “probable” categories within the Naranjo scoring criteria, 12 (10.3%) of charts in the preintervention group had a potential ADE, and 2 (4.3%) of charts in the postintervention group had a potential ADE (*p* = 0.35 by Fisher's exact test).

**Table 4:**
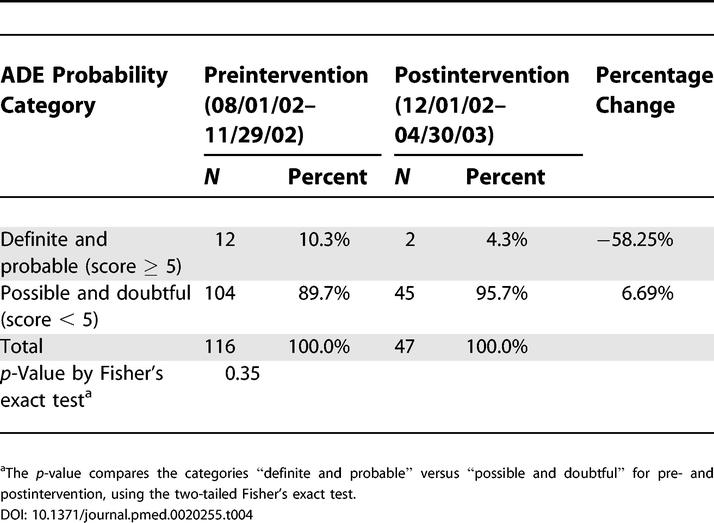
Adverse Drug Events Identified through Chart Review (Naranjo Scoring)

## Discussion

Health-care organizations are struggling with methods to improve the quality of care provided in a cost-efficient manner. Patient safety issues are primary concerns for health-care institutions and providers. Numerous examples have been published assessing the role of technology in assisting in these efforts [16–19]. The vast majority of data on technological interventions is focused on the inpatient hospital setting, often at tertiary care institutions, usually with house staff (physicians in training) programs, and rarely looks at commercially available applications [17,20–22]. Among geriatric patients, studies have shown a rate of 13.8 preventable ADEs per 1,000 person-years [23]. At Brigham and Women's Hospital, Boston, Massachusetts, implementation of inpatient CPOE led to an 81% decline in non-missed-dose medication error rates overall, and an 86% reduction in the intensive care units [24]. In an emergency department setting, computer-assisted prescriptions were more than three times less likely to contain errors than handwritten prescriptions [25].

In contrast, this study at Denver Health looked at a very specific type of clinical-decision support system: the use of a rules technology to prevent drug–laboratory adverse drug events. The clinical-decision support application and rule knowledge were both obtained from commercial vendors, and the CPOE application was a commercially available application. The setting was unique in that it was a primary-care outpatient setting. Furthermore, faculty physicians, as compared to training physicians, entered the majority of orders.

The clinical outcome portions of the study focused on assessing the effect of clinical-decision support systems on changing ordering behavior and, ultimately, in reducing ADEs among the patients. The study was not designed to have an adequate sample size to detect statistical difference in ADEs, although there were non-statistically fewer ADEs during the intervention phase. The rules did demonstrate a significant ability to change the ordering behavior of the provider. The effect was modest in halting the ordering of the medication and appeared to be limited to occasions in which the alert presented an abnormal laboratory value, with almost a doubling in order cessation. Still, the provider continued with ordering the medicine despite a warning message in the vast majority (91.7%) of the orders. This may be due to the providers deciding that the benefits of the medication far outweighed potential adverse effects on the associated laboratory abnormalities. In contrast, across all medication orders, and all categories of rules, ordering of the appropriate rule-associated laboratory test increased significantly (33% increase) with the presentation of an alert. The strongest effect was when providers where alerted to “missing” laboratory results (42% increase). Similar results have been found by Galanter et al. [26] when looking at automated safety alerts interactions between digoxin and potassium. In their study, checking for unknown potassium values increase from 9% to 57% after implementation of alerts. Likewise, in a community-based intervention by Hoch et al. [27], computerized alerts for missing potassium values sent the day after physicians had ordered a diuretic led to a 9.8% increase in potassium testing. Our study differed from these studies in that we looked at numerous medications across different therapeutic categories.

There was less of an effect on ordering behavior when the alert informed the provider of the existence of an abnormal laboratory value (23% increase in ordering of the test). This may imply that the cutoff values for the “abnormal” trigger were set too low, and that providers felt that repeating the laboratory test was not warranted given the degree of the abnormality. Further analysis, looking at the severity of the laboratory abnormality and correlating that to ordering behavior, may provide more insight to this issue.

There are various limitations to this study. The intervention only focused on a group of select drug–laboratory interactions and thus the results may not be generalizable to other types of interventions focusing on other patient-care issues. Further, the setting was in a single primary-care clinic outpatient setting within a large public-health integrated health-care delivery system and results may be different in other settings such as hospitals and private physician offices. The patient population served is primarily a lower income, minority-dominated (∼80% Hispanic), and medically underserved population. Different results may be obtained with a more affluent patient population. The study did not consider alert effectiveness based on the role of the provider. Further studies would be needed to determine if the provider role (i.e., staff physician, house staff, or nurse practitioner) may alter the effect of the alerts. Finally, as an evaluation of an intervention, the intervention was not randomized. Changes observed may have been occurring in the health-care environment irrespective of the intervention. The investigators are aware of no local or national initiatives to improve the care of these patients for the rule-associated conditions.

We conclude that with private–public entity collaboration, rules for drug–laboratory interactions can be encoded into computerized clinical applications in primary-care clinics within an integrated health-care delivery, safety-net institution. Further, with the use of clinical-decision support, providers will more often stop the ordering of medications when alerted to potential drug–laboratory interactions and will order more appropriate medication-associated laboratory tests. There may be an effect on ADEs. Future larger, more prolonged studies will help to determine the full relationship between automated alerts for drug–laboratory interactions and the related clinical outcomes of adverse drug events.

## Supporting Information

Protocol S1 Human Participant Approval (10 KB PDF).

Protocol S2 Initial Protocol (61 KB DOC).

**Patient Summary**

**Background**

All drugs have unwanted side effects (also known as adverse drug events), and when drugs are combined the chances of side effects increase. It is almost impossible for individual physicians to keep up to date with all possible drug effects. Increasingly, prescription orders and patient records are transmitted and stored on computers rather than being handwritten. As well as improving their legibility, computer writing of prescriptions also makes it possible to design programs that look at a patient's record when the prescription is written and that check for any possible problems.

**Why Was This Study Done?**

The authors wanted to investigate whether such a program could be used in an outpatient setting to change the behavior of physicians and ultimately to reduce the number of adverse drug reactions.

**What Did the Researchers Do and Find?**

In a single outpatient facility in Denver, Colorado, they designed a program to alert prescribers when one of five possible adverse events was likely to occur, or when the patient required further tests to establish whether the drug was likely to be safe. They tested the effect of the program by looking at what physicians did when the alerting system was switched off, and then when it was switched on. They found that it was possible to alter the behavior of prescribers by alerting them to possible problems; prescribers were more likely to stop a prescription or to order more tests when they were alerted. However, the study was too small to show for sure whether there was any true effect on adverse drug reactions.

**What Do These Findings Mean?**

Programs such as this one might be useful in alerting prescribers to potential problems with the drugs they are intending to prescribe. However, further work will need to be done to see if these programs can reduce the adverse events that patients experience, and whether they will work in other hospitals and clinics.

**Where Can I Get More Information Online?**

The US Web site MedlinePlus has a page of links on patient issues such as adverse reactions:

http://www.nlm.nih.gov/medlineplus/patientissues.html

The US Agency for Healthcare Research and Quality (AHRQ) provides a continuously updated, annotated, and carefully selected collection of patient safety news, literature, tools, and resources, including the Patient Safety Network:

http://psnet.ahrq.gov/

In the UK, the National Patient Safety agency site has information on many aspects of patient safety:

http://www.npsa.nhs.uk/

## Abbreviations

ADE: adverse drug event
CPOE: Computerized Physician Order Entry
