# Supplementary material for: The Effect of Automated Alerts on Provider Ordering Behavior in an Outpatient Setting
Source: PLoS Med. 2005 Sep 6;2(9):e255. doi: 10.1371/journal.pmed.0020255 (PMC1198038; doi:10.1371/journal.pmed.0020255)
Supplement: Supplementary file 1 [file pmed.0020255.sd001.pdf]

Colorado Multiple Institutional Review Board
13001 E. 17th Place
Building 500, Room N3214
Aurora, Colorado 80010-7238

Mailing Address:
Mail Stop F-490
P.O. Box 6508
Aurora, Colorado 80045-6508

(303) 724-1055 [Phone]
(303) 724-0990 [Fax]
www.uchsc.edu/comirb [Web]
comirb@uchsc.edu [E-Mail]
FWA#: FWA00005070

University of Colorado Hospital
Denver Health Medical Center
Veteran's Administration Medical Center
The Children's Hospital
University of Colorado Health Sciences Center
Colorado Prevention Center

Megan Jatko
A008/8701

01/14/2005

## Certificate of Exemption

Investigator: Sheri Eisert

Sponsor(s): AGENCY FOR HEALTHCARE RESEARCH AND QUALITY

Subject: **COMIRB Protocol 01-746**
**Initial Review (APP001)**
**2nd**

Title: THE EFFECT OF USING RULES TECHNOLOGY WITH PROVIDER ORDER ENTRY IN MEDICATION ERROR REDUCTION

Effective Date: **24 June 2002**

Expiration Date: **Exempt**

Exempt Category:

Includes: **Protocol - Investigator**

**This protocol qualifies for exempt status.** Periodic continuing review is not required. For the duration of your protocol, any change in the experimental design/content of this study must be approved by the COMIRB before implementation of the changes.

Any questions regarding the COMIRB action of this study should be referred to the COMIRB staff at 303-724-1055 or UCHSC Box F-490.

Ken Easterday, RPh, Co-Chair
Christopher Kuni, MD, Co-Chair
Norman Stoller, DMD, Co-Chair
Hans Neville, MD, Co-Chair
Dave Lawellin, PhD, Co-Chair
Douglas Ford, MD, Co-Chair
Steve Bartlett, RPh, Co-Chair
