# Supplementary material for: The Effect of Automated Alerts on Provider Ordering Behavior in an Outpatient Setting
Source: PLoS Med. 2005 Sep 6;2(9):e255. doi: 10.1371/journal.pmed.0020255 (PMC1198038; doi:10.1371/journal.pmed.0020255)
Supplement: Supplementary file 2 [file pmed.0020255.sd002.doc]

Task Order: Patient Safety and the Quality of Care: An Examination of Economic and Structural Characteristics, Working Conditions, and Technological Advances

# Technical Proposal

Denver Health

The Effect of Using Rules Technology with Provider Order Entry in Medication Error Reduction

August 17, 2001

**Introduction**

The burden of adverse drug events(ADEs) has been well documented in many reports.^[1],^^[2]^ Most of the studies on medical error reduction and the prevention of ADEs have occurred in the inpatient hospital setting.^[3]^ One of the few studies that has evaluated ADEs in an outpatient setting did not use clinical information system technology but used retrospective chart reviews and patient surveys.^[4]^ A recent meta-analysis suggested that in 1994 more than 1 million outpatients in the United States experienced an ADE that required admission to the hospital.^[5]^

As stated in a recent AHRQ report, medication errors are a frequent cause of ADEs and these errors can occur at any point in time during ordering, transcription, dispensing and administering medications.^[6]^ Most errors occur during the ordering and administration stages.

The Denver Health (DH) system is an ideal laboratory for the determination of the effectiveness of computerized clinical decision support systems and provider order entry on the potential reduction in medication errors. As an integrated delivery system serving as a safety-net, DH is comprised of 11 community health centers, 12 school-based clinics, 1 hospital, 4 dental clinics and the Department of Public Health. As a system, DH had approximately 20,000 inpatient admissions and over 600,000 outpatient visits during 2000. Over 50 percent of these visits were by Hispanics and over 40 percent of the visits were by the uninsured.

The DH information system is capable of incorporating rules within the Provider Order Entry (POE) system that will trigger a warning for a potential adverse drug event, not only in the inpatient setting but also the outpatient setting. As cited in the AHRQ report, *Making Health Care Safer: A Critical Analysis of Patient Safety Practices (www.ahrq.gov/clinic/ptsafety/)*, more research is necessary to determine the effectiveness of the use of computers to monitor providers’ drug orders for appropriateness and potential errors.

**Purpose**

This proposal addresses the evaluation of technical advances on patient safety and the quality of care. DH proposes to address the effects of the use of computerized decision support systems on prevention of adverse drug events within a computerized POE application. This project will be done in collaboration with Micromedex and Siemens Medical Solutions Health Services Corporation (see attached letter of support in Appendix A). The overarching purpose of this proposal is to determine the feasibility of using technology to improve safety related to prescribing medications in the outpatient setting. Specifically, the project will focus on adverse events related to medication use include hyper- and hypokalemia, nephrotoxicity, and thrombocytopenia. In addition this project will address provider satisfaction with the computerized decision support system within POE.

**Methodology**

In mid-October, 2001 POE will be implemented at one of DH’s larger outpatient clinics, Westside Clinic, that has about 50,000 outpatient visits per year. This clinic provides general medicine and specialty services, such as internal medicine, obstetrics/gynecology and pediatric services. As part of the implementation of POE in this clinic, rules will be implemented that will assist the provider in preventing potential adverse drug events.

The number of times a rule is fired and whether or not the medication is ordered when the rule is fired will be available within the rules engine audit log and from pharmacy records. Therefore, the number of potential averted adverse drug events will be tabulated based on whether or not the medication was ordered after a rule indicating a potential adverse event was displayed. In addition, the clinical course of patients for whom targeted medications are prescribed will be reviewed to identify development of targeted adverse drug events. Currently, comprehensive reporting mechanisms for detecting adverse drug events do not exist at DH.

The following rules will be evaluated:

1. Drug-induced Hypokalemia (ADE2): Warns of potential drug-induced hypokalemia.
2. Drug-induced Nephrotoxicity (ADE2): Detection of early drug-induced nephrotoxicity. If patient’s medication orders are available, it screens for more than 400 drugs known to cause renal toxicity.
3. Drug-induced Thrombocytopenia (ADE2): Detects thrombocytopenia and screens for a drug-induced cause.

Each of these rules requires certain data requirements (available from clinical data repository-Lifetime Clinical Record: LCR) as outlined in Appendix B. The rules will be defined by the Micromedex algorithms that link laboratory and medical record data to alert the provider of a potential adverse drug event if a particular drug is ordered.

The effects of the rules technology with provider order entry will be measured in two ways:

1. Concurrent comparisons of patient adverse drug events related to prescribed targeted medications will be made between intervention clinic (using POE) and a sampling of another clinics (not using POE).
2. Pre (9 months) and post (9 months) comparisons of patient adverse drug events related to targeted medications will be made at the intervention clinic. Adverse drug events will be defined as an occurrence of any of the targeted lab abnormalities (hyperkalemia, hypokalemia, nehrotoxicity-increasing creatinine, or thrombocytopenia) which develops in patients prescribed any of the related targeted medications. Collection of the incidence of these events will occur through ad-hoc reports written against the pharmacy and laboratory databases.

The effect of POE and rules on patients and ADEs will be measured by comparing the difference in ADE’s for those patients seen in a clinic with rules and POE to patients in a clinic without rules and POE. Patients who have been prescribed a defined list of medications will be targeted for study. The list of medications will be based on whether they would have the potential for creating an ADE related to medication use: hyper- and hypokalemia, nephrotoxicity, and thrombocytopenia. A comparable sample of patients at Westside clinic will be compared to a sample of similar patients from clinics where POE has not been implemented, and who have been prescribed the same medications. The rate of ADE’s will be compared between the two samples from both clinics over a 9 month period.

Provider behavior will be measured in two ways, one through whether the provider adhered to the rule and the other through a provider satisfaction survey. This information will only be collected the Westside clinic where POE will be implemented.

ADE aversion will be determined by the action taken by the provider or other clinician based on the alert or reminder. Adherence to the alert (e.g., cancellation of the specific order in response to an alert) is identified as ADE aversion. All medication-related alerts or reminders will be included in the denominator of this metric.

In addition, a provider satisfaction survey tool will be developed and administered 9 months after the intervention to all providers to assess issues related to compliance with the rules, and satisfaction with the intervention.

Further, review and approval by the Colorado Multiple Institution Review Board (COMIRB) will be obtained given the nature of the patient related intervention and storage/handling of some patient information and data. No consent form is anticipated for this study.

Siemens will provide the rules engine technology, provide testing and integration oversight, and facilitation on an as needed basis. Micromedex will provide rules knowledge content for items listed above, and review and facilitate the implementation phase to ensure the rules are meeting the expectations of their clinical guidelines. DH will provide project oversight and leadership, including the implementation of the rules at the test sites, and collection and analysis of data.

**Outcome Measures**

1. The study will acquire measure feasibility of using rules technology within a computerized POE application
2. Measurement of compliance with the rules recommendations will be made by comparing rules audit logs and prescription order data to determine how many potential adverse drug events occurred
3. Efficacy of prevention of adverse events will be monitored by reviewing incidence of targeted events of hypokalemia, hyperkalemia, nephrotoxicity, and thrombocytopenia
4. Careprovider satisfaction with the interaction with the rules technology application will be measured with a survey to be developed during the project.
5. Develop an implementation document that highlights steps, processes, barriers, and success factors for other institutions that may want to implement the use of rules technology in computerized POE projects, especially as it apples to the outpatient setting.

# Appendix B.

# Data Requirements for Micromedex Medical Logic Modules (MLM)

| **Medical Logic Module Name** | **Required Patient Data** | **Optional Patient Data** |
| --- | --- | --- |
| Drug-induced Hyperkalemia (ADE2)  Evoke: ODT  Warns of potential drug-induced hyperkalemia. | Medication orders (can be optional)  Date of birth  Serum potassium | None |
| Drug-induced Hypokalemia (ADE2)  Evoke: ODT  Warns of potential drug-induced hypokalemia. | Medication orders (can be optional)  Date of birth  Serum potassium | None |
| Drug-induced Nephrotoxicity (ADE2)  Evoke: ODT  Detection of early drug-induced nephrotoxicity. If patient’s medication orders are available, it screens for more than 400 drugs known to cause renal toxicity. | Date of birth  At least one serum creatinine | Patient’s medication orders |
| Drug-induced Thrombocytopenia (ADE2)  Evoke: ODT  Detects thrombocytopenia and screens for a drug-induced cause. | At least one platelet count | Patient service and/or location  Medication orders |
| Health Maintenance Rules (Wellness)  Evoke: ODT  Screening reminders for outpatient clinics. Includes screening reminders for immunizations, cholesterol, blood pressure and the following cancers: colorectal, breast, and cervical. | Date of birth, race, gender,  Systolic and diastolic blood pressures  Immunization history, laboratory data  Problem list and medical history | None |

## Abbreviations:

ODT	On demand trigger. An MLM that contains user definable arguments that control the functionality of the associated clinical rule.

POE	Physician or provider order entry.

## MLM Classification by Clinical Objective:

ADE1	Primary prevention of an adverse drug event.

ADE2	Secondary prevention by early detection of an adverse drug event.

CPG	Clinical practice guideline – provides assistance with clinical guidelines.

IVPO	Conversion from intravenous to the oral route of administration. Some MLMs provide suggestions for oral substitutes.

RX	Drug use guideline – provides alerts or reminders regarding pharmacotherapy guidelines.

1. Institute of Medicine. To err is human: building a safer health system. Washington: National Academy Press; 1999.
2. Thomas EJ et. al. Incidence and types of adverse events and negligent care in Utah and Colorado. Med Care 2000;38(3):261-71..
3. Bates DW, Teich JM, Lee J, Seger D, Kuperman GJ, Ma’Luf N, Boyle D, Leape L. The impact of computerized physician order entry on medication error prevention. J Am Med Inform Assoc. 1999:4:313-321.

   Teich JM, Merchia PR, Schmiz JL, Kuperman GJ, Spurr CD, Bates DW. Effects of computerized physician order entry on prescribing practices. Arch Intern Med. 2000;160:2741-2747.

   Kaushal R, Bates DW, Landrigan C, McKenna KJ, Clapp MD, Federico F, Goldmann DA. Medication errors and adverse drug events in pediatric inpatients. JAMA. 2001:285:2114-2120.

   Bates DW, Leape LL, Cullen DJ, Laird N, Peterson LA, Teich JM, Burdick E, Hickey M, Kleefield S, Shea B, Vander Vliet M, Seger DL. Effect of computerized physician order entry and a team intervention on prevention of serious medication errors. JAMA. 1998;280:1311-1316.

   Raschke RA, Gollihare B, Wunderlich TA, Guidry JR, Leibowitz AI, Peirce JC, Lemelson L, Heisler MA, Susong C. A computer alert system to prevent injury from adverse drug events. JAMA. 1998;280:1317-1320.
4. ^?^ Gandhi TK, Bursti HR, Cook EF, Puopolo AL, Haas JS, Brennan TA, Bates DW. Drug complications in outpatients. J Gen Intern Med 2000;15:149-154.
5. Lazarou J, Pomeranz BH, Corey PN. Incidence of adverse drug reactions in hospitalized patients: a meta-analysis of prospective studies. JAMA. 1998;279:1200-5.
6. Agency for Healthcare Research and Quality. Reducing and preventing adverse drug events to decrease hospital costs. Research in Action, March 2001, Issue #1.
